# Metformin and sitAgliptin in patients with impAired glucose tolerance and a recent TIA or minor ischemic Stroke (MAAS): study protocol for a randomized controlled trial

**DOI:** 10.1186/s13063-015-0882-z

**Published:** 2015-08-05

**Authors:** Elizabeth Osei, Susanne Fonville, Adrienne A. M. Zandbergen, Paul J. A. M. Brouwers, Laus J. M. M. Mulder, Hester F. Lingsma, Diederik W. J. Dippel, Peter J. Koudstaal, Heleen M. den Hertog

**Affiliations:** Department of Neurology, Erasmus Medical Center University Hospital, Rotterdam, The Netherlands; Department of Neurology, Medisch Spectrum Twente, PO Box 50 000, Enschede, 7500 KA The Netherlands; Department of Internal Medicine, Ikazia Hospital, Rotterdam, The Netherlands; Department of Neurology, Ikazia Hospital, Rotterdam, The Netherlands; Department of Public Health, Erasmus Medical Center University Hospital, Rotterdam, The Netherlands

**Keywords:** Stroke, Impaired glucose tolerance, Anti-diabetic drugs

## Abstract

**Background:**

Impaired glucose tolerance is present in one third of patients with a TIA or ischemic stroke and is associated with a two-fold risk of recurrent stroke. Metformin improves glucose tolerance, but often leads to side effects.

The aim of this study is to explore the feasibility, safety, and effects on glucose metabolism of metformin and sitagliptin in patients with TIA or minor ischemic stroke and impaired glucose tolerance. We will also assess whether a slow increase in metformin dose and better support and information on this treatment will reduce the incidence of side effects in these patients.

**Methods/Design:**

The Metformin and sitAgliptin in patients with impAired glucose tolerance and a recent TIA or minor ischemic Stroke trial (MAAS trial) is a phase II, multicenter, randomized, controlled, open-label trial with blinded outcome assessment. Non-diabetic patients (*n* = 100) with a recent (<6 months) TIA, amaurosis fugax or minor ischemic stroke (modified Rankin scale ≤ 3) and impaired glucose tolerance, defined as 2-hour post-load glucose levels between 7.8 and 11.0 mmol/L after repeated standard oral glucose tolerance test, will be included. Patients with renal or liver impairment, heart failure, chronic hypoxic lung disease stage III–IV, history of lactate acidosis or diabetic ketoacidosis, pregnancy or breastfeeding, pancreatitis and use of digoxin will be excluded. The patients will be randomly assigned in a 1:1:2 ratio to metformin, sitagliptin or “no treatment.” Patients allocated to metformin will start with 500 mg twice daily, which will be slowly increased during a 6-week period to a twice daily dose of 1000 mg. Patients allocated to sitagliptin will be treated with a daily fixed dose of 100 mg. The study has been registered as NTR 3196 in The Netherlands Trial Register. Primary outcomes include percentage still on treatment, percentage of (serious) adverse events, and the baseline adjusted difference in 2-hour post-load glucose levels at 6 months.

**Discussion:**

This study will give more information about the feasibility and safety of metformin and sitagliptin as well as the effect on 2-hour post-load glucose levels at 6 months in patients with TIA or ischemic stroke and impaired glucose tolerance.

**Trial registration number:**

NTR3196, Date of registration: 15 December 2011.

## Background

Impaired glucose tolerance, an intermediate metabolic state between normal glucose tolerance and diabetes mellitus, is present in about a third of patients with transient ischemic attack (TIA) or ischemic stroke [1–4], and is associated with a two-fold risk of recurrent stroke [5]. The mechanisms underlying this association are not fully understood, but include insulin resistance, endothelial dysfunction, dyslipidemia, chronic inflammation, procoagulability, and impaired fibrinolysis [6–8].

**Fig. 1:**
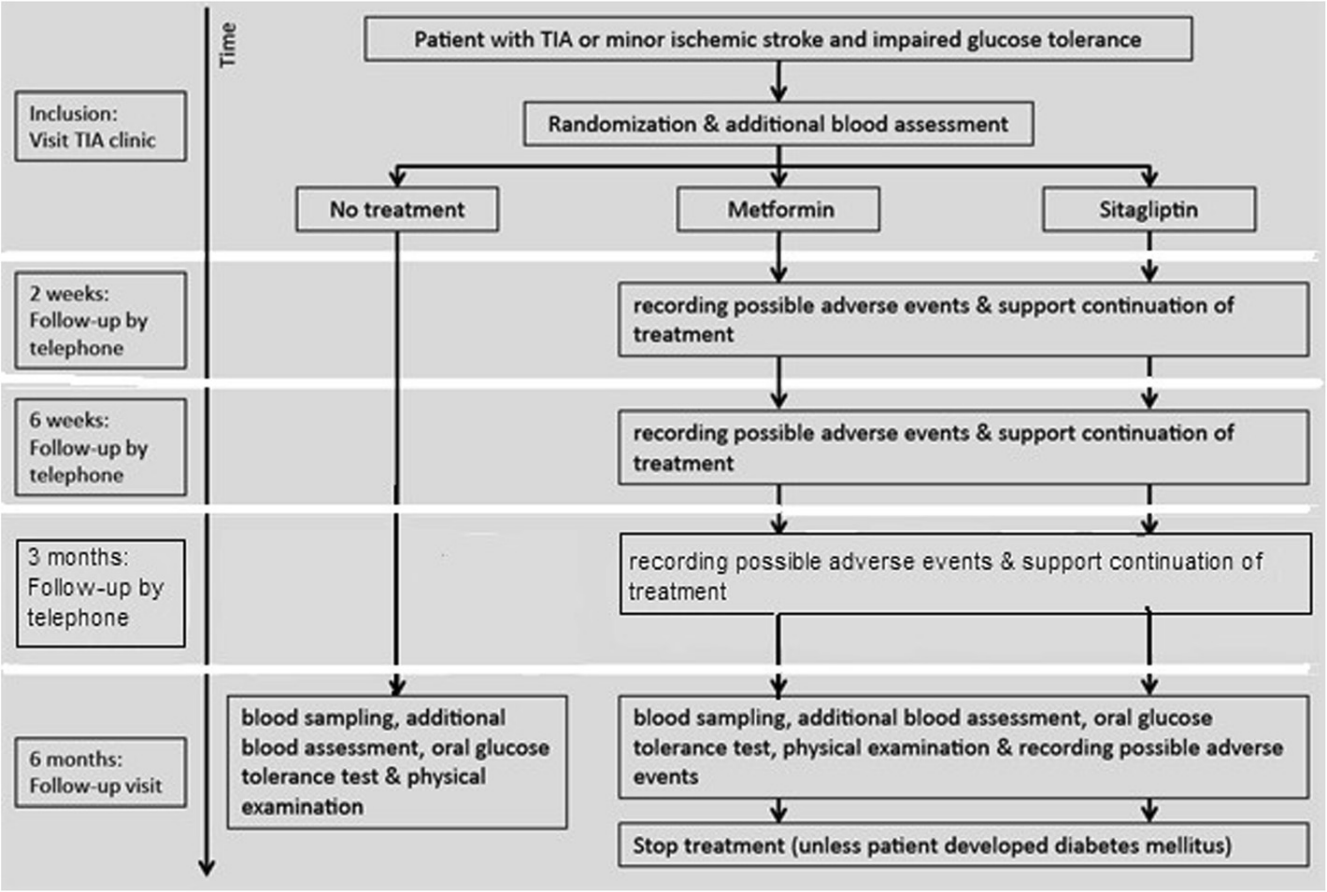
Flowchart study design

Pharmacological interventions reduce the rate of progression to type 2 diabetes by 10–60 % in people with impaired glucose tolerance [9–12]. Lifestyle interventions are likely to be at least as effective as drug treatment [9, 12], but are often difficult to carry out successfully, and lifestyle advice needs to be reinforced on a regular basis.

There is no clear evidence that tight glycemic control reduces the risk of stroke in patients with diabetes or impaired glucose tolerance. A recent meta-analysis on glucose-lowering pharmacological interventions in patients with impaired glucose tolerance found no beneficial effects on all-cause mortality or death due to major cardiovascular events, with the possible exception of stroke [13].

In the UK Prospective Diabetes Study, however, metformin therapy or intensive treatment with sulphonylurea or insulin seems to be associated with fewer cardiovascular events in newly-diagnosed type 2 diabetics [14]. Furthermore, a large randomized placebo-controlled trial found that metformin reduces macrovascular complications when added to insulin treatment in type 2 diabetes [15].

However, metformin had no effect on the carotid intima media-thickness or carotid plaque in stroke patients [16]. Also, metformin was not more effective in preventing myocardial infarction than other intensive therapy. Furthermore, metformin added to sulphonylurea therapy was associated with an increased risk for diabetes-related deaths and all-cause mortality [14]. Metformin is regarded as one of the most effective drugs for treating type 2 diabetes. Recent basic research reveals that suppression of hepatic gluconeogenesis by inhibiting mitochondrial glycerophosphate dehydrogenase is the main underlying mechanism of metformin's blood glucose-lowering effect [17].

Metformin is recommended as first-line treatment in type 2 diabetes mellitus, and is cheap as compared to the newer antidiabetic drugs. Our recent findings suggest that metformin treatment is safe in patients with TIA or ischemic stroke and impaired glucose tolerance, and probably leads to improved glucose tolerance [18]. However, 50 % of the patients experienced gastrointestinal side effects resulting in permanent discontinuation in 25 %. Slower increase in dose of metformin and better information and support on the temporary nature of the side effects might prevent the high incidence of side effects and discontinuation of treatment respectively.

Novel drugs, like selective dipeptidyl peptidase-4 (DPP-4) inhibitors for type 2 diabetes, might have fewer side effects than metformin and other anti-diabetic medication. They target primarily postprandial glucose, which is more closely associated with carotid intimal thickness and atherosclerosis risk factors than fasting plasma glucose or glycosylated hemoglobin A1 (HbA1c) levels [19]. Sitagliptin, a selective DPP-4 inhibitor, improves glycemic control and *β*-cell function and has a safety profile similar to placebo, with low risk of gastrointestinal side effects [20, 21]. Also, it is associated with weight loss and a lower risk on hypoglycemia [20, 21]. However, some recent trials have shown that DPP-4 inhibitors had no effect on adverse cardiovascular outcomes in patients with type 2 diabetes [19].

The aim of the study is to explore the feasibility, safety, and effect on glucose metabolism of both metformin and sitagliptin in patients with TIA or minor ischemic stroke and impaired glucose tolerance. Also, we will assess whether a slow increase in dose of metformin and better support and information on this treatment will reduce the incidence of side effects in these patients.

## Methods

### Design

We conduct a phase II, multicenter Prospective, Randomized, Open-label, Blinded End-point (PROBE) trial of standard care plus metformin or sitagliptin, as compared with standard care without antidiabetic treatment.

### Patient population – inclusion and exclusion criteria

All adult patients attending the TIA outpatient clinic or admitted to the stroke unit in 3 hospitals in the Netherlands with TIA, amaurosis fugax or minor ischemic stroke (defined as a modified Rankin scale (mRS) [22] score of 3 or less) within the previous 6 months, and impaired glucose tolerance, defined as 2-hour post-load glucose levels between 7.8 and 11.0 mmol/L [23] after standard oral glucose tolerance test (OGTT) [24], will be invited to participate in the trial by their neurologist.

The diagnosis of TIA (symptoms < 24 hours) or ischemic stroke will be made by a neurologist by standard guidelines. An ischemic stroke or TIA will be defined as an episode of neurological dysfunction caused by focal cerebral or retinal infarction [25]. All patients will undergo a computed tomography (CT) scan of the brain, electrocardiogram, carotid ultrasound imaging and blood investigations, which also includes the lipid profile.

The OGTT will be repeated after 2–6 weeks to rule out laboratory error and the acute phase effect. If the second OGTT confirms the diagnosis of impaired glucose tolerance, and all the selection criteria are fulfilled, the patient will be asked for written informed consent by the investigators. So, written informed consent will be obtained by all participants. Inclusion and exclusion criteria are shown in Table 1.

**Table 1:** Inclusion and exclusion criteria

Inclusion criteria:
Age ≥ 18 years
TIA, amaurosis fugax or minor ischemic stroke (mRS ≤ 3)
Symptom onset < 6 months
Impaired glucose tolerance (2-hour post-load glucose level between 7.8 and 11.0 mmol/L) in 2 consecutive measurements
Exclusion criteria:
Diabetes mellitus
History of diabetic ketoacidosis
Symptoms of type 1 diabetes
Signs of renal impairment (creatinine of 135 μmol/L or higher for men, and 110 μmol/L or higher for women)
Known liver disease or disturbed liver function tests (alanine amino transferase, aspartate amino transferase, alkaline phosphatase, or γ-glutamyl transferase increased to more than twice the upper limit of typical values)
History of lactic acidosis
Heart failure requiring pharmacological therapy
Pancreatitis
Chronic hypoxic lung disease stages III–IV
Digoxin use
Pregnancy or breastfeeding

*mRS* modified Rankin Scale, *TIA* transient ischemic attack

### Randomization, blinding and treatment allocation

Patients will be randomized by the investigators to receive either open-label metformin or sitagliptin or “no treatment” in a 1:1:2 ratio for 6 months. The randomization process will be available online by means of a list generated by computer before the start of the trial. Treatment allocation will be only possible after registration in the database. From this moment, it will not be possible to remove a patient from the database. The list with information regarding the treatment allocation will be kept separate from the study database. An independent statistician, who otherwise will not be involved in the study, will provide the list. The statistician will report unblinded data to the Data Safety and Monitoring Board (DSMB) for evaluation and interim analysis, for monitoring the safety and progression of the trial. The steering committee will be kept unaware of these results unless necessary (as judged by the DSMB), and the code will not be broken until the last patients have completed the 6-months of follow-up.

Irrespective of treatment allocation, patients will receive optimal standard care from the neurologist, including antithrombotic and antihypertensive agents as well as cholesterol- lowering drugs, where appropriate [26]. In addition, a stroke nurse specialist will provide general lifestyle advice including healthy diet, stopping smoking, and regular physical exercise. These interventions will also be monitored by the investigators through the follow-up contacts.

### Intervention

Patients will be randomly allocated to open-label metformin or sitagliptin or “no treatment” for a 6-month period. The treatment assignment is concealed. Patients allocated to metformin will start with 500 mg twice daily, which will be slowly increased in a 6-week period to 1000 mg twice daily (week 1: 2 times 500 mg, week 3: 2 times 850 mg, week 7: 2 times 1000 mg). If there are unmanageable side effects at an increased dose, the lower dose will be resumed and the increase will be tried again the next week. Patients allocated to sitagliptin will be treated with a daily fixed dose of 100 mg.

### Study procedures

All patients will be assessed at baseline, and at 6 months. At baseline, data on clinical features of TIA or ischemic stroke, demographic data, medical history, vascular risk factors, and medication use will be obtained.

At the follow-up visit, patients will be asked to complete a single questionnaire to determine compliance and nature of any of the side effects of the study medication. Patients will also be contacted by telephone for recording of possible adverse events and to support continuation of treatment at 2 weeks, 6 weeks, and 3 months after inclusion. If necessary (e.g. in case of unmanageable side effects), the number of telephone contacts and or follow-up visits will be intensified. Before randomization and at 6 months, all patients will undergo an OGTT with 75 g of glucose. Fasting glucose levels, body mass index (BMI), waist circumference, blood pressure and lipid profile will be assessed at baseline, and at 6 months (Fig. 1).

Patients will be excluded from other trials which involve any secondary prevention treatment of stroke like new medication or lifestyle adjustments. The final raters who will assess the outcome measures will be blinded for treatment allocation.

### Primary outcomes

The primary outcomes are tolerability of metformin and sitagliptin, assessed as the number of patients still on treatment after 6 months, the number of adverse events and serious adverse events, and the baseline adjusted difference in 2-hour post-load glucose levels.

### Secondary outcomes

Secondary outcomes are the effect of metformin and sitagliptin on fasting plasma glucose levels at 6 months, the percentage of patients with normal glucose tolerance at 6 months and on the BMI and waist circumference at 6 months.

### Data safety monitoring board

The DSMB is composed of independent experts in the field of statistics, neurology, and vascular internal medicine. It monitors the progress and safety of the trial, and performs an interim analysis. Based on this information, they advise the steering committee on pre-specified grounds, as formulated by the DSMB.

### Sample size

We expect that 50 % of patients on metformin will experience side effects during follow-up. Assuming a difference of 40 % in side effects between patients on metformin and patients in the control group, and a difference of 30 % between patients on metformin and patients on sitagliptin, 100 patients will have a power of 80 % to detect a significant (α = 0.05) difference in side effects.

A sample size of 100 patients, calculated by a statistician, (25 on metformin, 25 on sitagliptin, and 50 in the control group) will provide a power of 80 %, to detect a difference of 8 % in 2-hour post-load glucose level after 6 months between treatment groups, assuming a significance level of α = 0.05 and a mean glucose level of 9.0 mmol/L in the control group, with a standard deviation of 1.0 mmol/L.

### Statistical analyses

Analyses will be done by intention-to-treat and all patients who are randomly assigned to treatment will be included in the pre-specified analyses. The effect in each of the two treatment groups will be compared to the control group separately.

We will estimate the baseline adjusted differences in mean 2-hour post-load glucose levels and fasting glucose levels between treatment groups with 95 % confidence intervals (CIs) with univariable linear regression. Adjustments will be made with a multivariable linear regression model that will include the following factors: age, sex, time to treatment, and baseline waist. Similar analyses will be performed to study the effect of treatment with metformin or sitagliptin on BMI and waist circumference. We will compare the percentage of patients still on treatment after 6 months, the incidence of (serious) adverse events and percentage of patients with a normal glucose tolerance at 6 months between treatment groups with chi-square test.

No adjustment for multiplicity will be done due to the explanatory nature of a phase II trial.

### Ethical approval

The Medical Ethical Trial Commissions (METC) of both the Erasmus Medisch Centrum in Rotterdam and Medisch Spectrum Twente in Enschede gave approval for this trial.

## Discussion

Impaired glucose tolerance is present in up to one third of patients with TIA or stroke and is associated with a two-fold increased risk of recurrent stroke. The increased risk of impaired glucose tolerance could be due to the impaired lipid profile of these patients. However, there is an independent association of impaired glucose tolerance with cardiovascular diseases. Intensive glucose control with oral antidiabetic drugs reduces the rate of progression to type 2 diabetes in patients with impaired glucose tolerance [9–12]. Whether pharmacotherapeutic intervention reduces the risk of cardiovascular events in patients with TIA or minor ischemic stroke (who are often older with more co-medication) and impaired glucose tolerance is unknown. Our recent study (LIMIT) has shown that metformin treatment is safe and improves glucose tolerance in these patients, but often leads to gastrointestinal side effects [18].

The rationale for choosing metformin and sitagliptin is as follows: metformin is recommended as first-line treatment in type 2 diabetes, is cheap, and widely used in The Netherlands. However, in the LIMIT trial metformin caused frequent gastrointestinal side effects and consequently discontinuation of drug adherence in a large percentage of the patients [18]. When slow increase in dose of metformin and better support and information on this treatment proves to reduce the incidence of side effects in these patients, metformin will be a cheap, easy and widely used drug to improve glucose tolerance in a large proportion of stroke patients. Sitagliptin has proven to be equally effective and safe as metformin, with improvement of glycemic control by lowering HbA1c levels, and a lower risk of gastrointestinal side effects and hypoglycemia [20, 21]. This combined with the once daily, fixed dose makes it a patient-friendly drug and is more prone to good adherence. However, the costs of sitagliptin are more than 16 times higher compared to metformin. To compare the effect in both treatment groups with the control group separately, a randomization ratio of 1:1:2 was chosen.

Although a double-blind, placebo-controlled designed trial would have been superior, the current trial has a PROBE design. The main reason is that the former comes with greater costs. Potential limitations are therefore the lack of a placebo group and its open design, which can give an increased risk on performance bias. However, outcome assessment will be blinded for treatment allocation. The final raters who will assess the outcome will be blinded for treatment allocation. And the design will resemble the effect in clinical practice after implementation.

Although lifestyle intervention is equally effective in lowering glucose levels as glucose- lowering drugs, it is hard to sustain [9, 12]. If we can prove with the MAAS trial that metformin and/or sitagliptin are safe and feasible in lowering glucose levels, a phase III trial is necessary to investigate the effect on the incidence of recurrent stroke and other cardiovascular complications, to improve secondary prevention in these patients. In this phase III trial, patients with a TIA or minor ischemic stroke and impaired glucose tolerance will receive standard care and be allocated to metformin or sitagliptin, depending on the results of this phase II-trial, and one of these will be compared to placebo.

## Trial status

The MAAS trial started in January of 2014. Thirty-two patients have been included so far.

## Abbreviations

BMI: body mass index
CI: confidence interval
CT: computed tomography
DPP-4: dipeptidyl peptidase-4
DSMB: Data Safety and Monitoring Board
HbA1c: glycosylated hemoglobin A1
MAAS trial: Metformin and sitAgliptin in patients with impAired glucose tolerance and a recent TIA or minor ischemic Stroke trial
METC: Medical Ethical Trial Commissions
mRS: modified Rankin Scale
OGTT: oral glucose tolerance test
PROBE: Prospective Randomized Open, Blinded End-point, TIA: transient ischemic attack
